# Lipid-Coated Nanocrystals as a Tool for Improving the Antioxidant Activity of Resveratrol

**DOI:** 10.3390/antiox11051007

**Published:** 2022-05-20

**Authors:** Monica Argenziano, Irfan Aamer Ansari, Elisabetta Muntoni, Rita Spagnolo, Anna Scomparin, Roberta Cavalli

**Affiliations:** Department of Scienza e Tecnologia del Farmaco, University of Turin, Via P. Giuria 9, 10125 Turin, Italy; monica.argenziano@unito.it (M.A.); irfanaamer.ansari@unito.it (I.A.A.); elisabetta.muntoni@unito.it (E.M.); rita.spagnolo@unito.it (R.S.); anna.scomparin@unito.it (A.S.)

**Keywords:** *trans*-resveratrol, nanocrystals, lipid coating, oral bioavailability

## Abstract

*Trans*-resveratrol, a polyphenolic phytoalexin found in various plant sources, has been the focus of increasing attention in recent years because of its role in the prevention of many human diseases, and particularly because of its antioxidant properties. However, the in vivo effect of *trans*-resveratrol after oral administration is negligible when compared to its efficacy in vitro, due to its low bioavailability. Moreover, it presents stability issues as it is an extremely photosensitive compound when exposed to light. This work aims to develop lipid-coated nanocrystals in order to improve the antioxidant activity and bioavailability of *trans*-resveratrol. Lipid-coated *trans*-resveratrol nanocrystals with sizes lower than 500 nm, spherical shapes and smooth surfaces were obtained via a milling method. They showed a faster dissolution rate than the coarse *trans*-resveratrol powder. The antioxidant properties of *trans*-resveratrol were not impaired by the milling process. The in vivo pharmacokinetics of lipid-coated *trans*-resveratrol nanocrystals were evaluated after oral administration to rats, with a commercial Phytosome^®^ formulation being used for comparison purposes. An increase in the *trans*-resveratrol area under the curve was observed and the lipid-coated nanocrystal formulation led to an enhancement in the oral bioavailability of the compound.

## 1. Introduction

**Figure 1:**
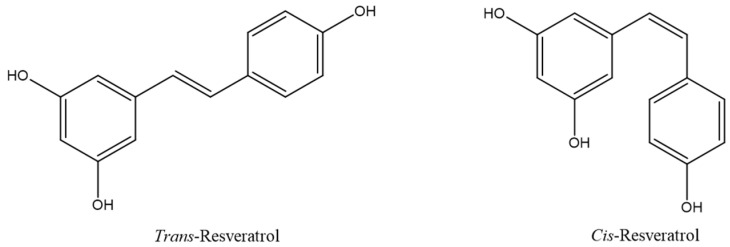
Chemical structures of *trans*-resveratrol and *cis*-resveratrol.

Resveratrol (3,5,4′-trihydroxy-*trans*-stilbene, RV) is a polyphenolic phytoalexin, found in a variety of plant species. Phytoalexins are low molecular weight secondary metabolites that are produced by plants as a defense response to exogenous stress factors, such as injury, microbial infections and ultraviolet (UV) irradiation. RV presents two geometric isomeric forms, *cis* and *trans* (Figure 1).

The *trans*-isomer is predominant and possesses biological activity, but can be easily isomerized to the *cis*-isomeric form after exposure to sunlight or UV light [1]. RV is an antioxidant compound, mainly due to the presence of hydroxylic groups in its backbone, which are involved in mechanisms that can reduce reactive oxygen species (ROS) and free radicals [2]. In addition, it shows the ability to upregulate the expression of cellular defensive genes against oxidative stress by increasing the biosynthesis of endogenous antioxidants [3]. RV has been reported to play an important role in the prevention and therapy of cardiovascular diseases and cancers, especially thanks to its antioxidant properties [4,5]. However, the administration of RV remains challenging due to its low oral bioavailability [6].

RV, in the manner of many other active ingredients of natural origin, has a low solubility (0.03 g/L) and hydrophobic character (LogP~3.4), and can be classified into Class II of the Biopharmaceutical Classification System (BCS) [7]. In particular, its hydrophobicity means that dissolution is the rate-limiting step for in vivo absorption. It therefore shows a poor bioavailability that is associated with a rapid and extensive pre-systemic metabolism and high systemic clearance [8].

Several formulation strategies have been proposed to improve the solubility, chemical stability and oral bioavailability of RV and these include the development of polymeric nanoparticles, liposomes, solid lipid nanoparticles, solid dispersions, microemulsions and cyclodextrin-based formulations [9,10,11,12,13,14]. In addition, resveratrol-based inorganic nanoparticles (i.e., mesoporous silica nanoparticles, gold nanoparticles and iron oxide nanoparticles) have been also studied [15,16,17].

Interestingly, nanocrystal (NC) formulations have been proposed as a means to improve the oral bioavailability of poorly soluble drugs that are mainly limited by their dissolution rate and inadequate solubility. This is possible because of the special features of drug nanocrystals, which include increased saturation velocity, enhanced dissolution rate and better adhesiveness to surfaces/cell membranes compared to the micro-sized drug powders [18,19,20]. A considerable number of nanocrystal-based pharmaceuticals are currently available on the market [21,22].

The formulation of RV as nanocrystals and their administration via different routes (i.e., intravenous, oral and dermal) have already been described in the literature [23]. For example, Sinico and colleagues have prepared a stable RV nanocrystal nanosuspension using the wet media milling method and by adding Poloxamer 188 and Tween 80 as stabilizers in order to improve RV dermal delivery [24]. In other research, RV nanocrystals have been manufactured via probe sonication and by exploiting various stabilizers, such as TPGS, Lecithin and Pluronic F127, leading to an enhancement in the oral bioavailability of RV [25].

The use of lipid formulations, such as liposomes, emulsions and solid lipid nanoparticles (SLN), is another valuable technological approach for improving the therapeutic efficacy of poorly bioavailable drugs [26]. Lipid systems have been extensively studied as a means for the delivery of natural compounds in order to increase solubility and stability, enhance drug bioavailability and offer control over absorption and distribution profiles [27,28]. Indeed, a RV-phospholipid complex (Phytosome^®^) is on the market [29].

The goal of this study is to improve the antioxidant activity and oral bioavailability of *trans*-resveratrol by combining the benefits of nanocrystals and lipid formulations. The development and the in vitro characterization of resveratrol lipid-coated nanocrystals are reported in this paper. In addition, we present an in vivo evaluation of the pharmacokinetic parameters following the oral administration of the formulation to rats.

## 2. Materials and Methods

### 2.1. Materials

All the reagents were obtained from Sigma-Aldrich (Sigma-Aldrich Chemical, St. Louis, MO, USA), unless otherwise specified. Methanol and acetic acid were of HPLC grade. Ultrapure water was obtained using a 1–800 Millipore system (Merck Millipore, Molsheim, France). Epikuron 200^®^ (Dipalmitoyl phosphatidylcholine 92% and accompanying phospholipids) was provided by Cargill (Wayzata, MN, USA). Commercial resveratrol Phytosome^®^ was obtained from Indena (Milan, Italy).

### 2.2. Preparation of Trans-Resveratrol Nanocrystals

*Trans*-resveratrol nanocrystals (RV-NC) were prepared via pearl milling (PM 100, Retsch, Haan, Germany), which was operated for 45 min at a rate of 400 rpm. For this purpose, a weighted amount of RV powder was filled into the milling chamber that had been charged with milling pearls (3 mm stainless-steel grinding balls). The lipid-coated nanocrystals (LC RV-NC) were obtained by adding soybean lecithin (Epikuron 200^®^) to the milled RV powder at a weight ratio of 1:1 and then performing a second milling step using pearl milling (PM 100, Retsch) for 30 min at a rate of 250 rpm. 

Aqueous nanosuspensions of RV-NC and LC RV-NC were prepared by dispersing the NC powders in water. A RV suspension was made as a control by grinding the coarse RV powder in a mortar and then suspending it in water that contained hydroxypropyl cellulose (0.5% *w*/*v*).

### 2.3. In Vitro Characterization of Trans-Resveratrol Nanocrystals

#### 2.3.1. Physico-Chemical Parameter Determination

The average diameter, polydispersity index and Zeta potential of the *trans*-resveratrol formulations were determined by dynamic light scattering (DLS) using a 90 plus instrument (Brookhaven Instruments Corporation, New York, NY, USA), at a fixed scattering angle of 90° and a temperature of 25 °C. The samples were diluted in filtered distilled water prior to measurements. Each reported value is the average of ten measurements of three different formulation batches.

#### 2.3.2. Scanning Electron Microscopy (SEM) Analysis

The morphology of *trans*-resveratrol nanocrystals, either lipid-coated or uncoated, was determined by scanning electron microscopy analysis (SEM; Stereoscan 410, Leica, Wetzlar, Germany). The control, mortar-ground, coarse RV powder was evaluated by SEM analysis. The powder was mounted onto stubs using double sided adhesive tape. The samples were sputter coated with aurum in an argon atmosphere and examined at an accelerating voltage of 15 kV.

#### 2.3.3. Differential Scanning Calorimetry (DSC)

The thermal analysis of *trans*-resveratrol nanocrystals, both the lipid-coated and uncoated counterparts, was carried out using a DSC/7 differential scanning calorimeter (Perkin-Elmer, Branford, CT, USA) that was equipped with a TAC 7/DX instrument controller and the Pyris Manager program. The instrument was calibrated with indium for melting point and heat of fusion before analyses took place. A heating rate of 10 °C per minute was used in the 25–280 °C temperature range. The thermal behavior was studied by heating roughly 3 mg of the samples in standard aluminum sample pans (Perkin-Elmer), with an empty aluminum pan being used as the reference standard. Analyses were carried out under nitrogen purge and triple runs were performed for each sample.

#### 2.3.4. Fourier Transform Infra-Red Spectroscopy (FTIR)

The coarse RV powder and RV nanocrystals, both uncoated and lipid-coated, were subjected to Fourier transform infra-red spectroscopy (FTIR) studies using the potassium bromide disc method on a Perkin Elmer system 2000 FTIR Spectrophotometer in the region of 4000–650 cm^−1^. Data acquisition was carried out using Spectrum software version 5.0.2 Perkin Elmer Corporation (Perkin Elmer Inc., Hopkinton, MA, USA). 

### 2.4. Trans-Resveratrol Quantitative Determination

The quantitative determination of *trans*-resveratrol was accomplished on a HPLC system that consists of a pump (Perkin Elmer PUMP 250B) equipped with a spectrophotometer detector (LC 95). Analyses were performed on an Agilent TC C18 column (250 mm × 4.6 mm, 5 µm). The mobile phase was a mixture of methanol and acetic acid 0.5% (48:52 *v*/*v*) that was degassed and pumped through the column at a flow rate of 1.0 mL/min. Ultraviolet detection was set at 303 nm.

### 2.5. In Vitro Dissolution Studies

A comparison between the dissolution behavior of coarse *trans*-resveratrol powder, RV Phytosome^®^ and nanosized *trans*-resveratrol (RV-NC and LC RV-NC) was carried out in simulated intestinal fluid (pH 6.8). Weighed amounts (5 mg) of coarse RV powder and RV nanocrystals, both uncoated and lipid-coated, were suspended in 50 mL of simulated intestinal fluid (pH 6.8). The vials were placed under magnetic stirring at room temperature.

At a pre-selected time, 250 µL samples were withdrawn and replaced with the same amount of fresh dissolution medium. After centrifugation and filtration through a membrane filter (0.22 μm), the samples were analyzed by HPLC for RV quantitative determination, as described in the previous paragraph (Section 2.4).

### 2.6. Stability Studies

The physico-chemical stability of the *trans*-resveratrol nanocrystals (RV-NC and LC RV-NC) that were suspended in water was investigated in vitro. The morphology, particle size and surface charge of the RV formulations were evaluated over time.

### 2.7. Antioxidant Activity Evaluation

#### 2.7.1. DPPH Free Radical Scavenging Activity

The antioxidant activity of resveratrol formulations was evaluated by measuring their ability to scavenge 2,2-diphenyl-1-picrylhydrazyl (DPPH). DPPH is a lipophilic free radical that has a purple color in solution and UV absorption at a wavelength of 517 nm. When DPPH undergoes a redox reaction with an antioxidant, it turns yellow with a decrease in absorbance at 517 nm.

To perform the DPPH free radical scavenging assay, 100 μL of each sample (coarse RV suspension, RV Phytosome^®^ suspension, RV-NC and LC RV-NC nanosuspensions at different concentrations) were added to a DPPH methanolic solution (900 μL, 0.1 mM) and incubated at room temperature for 30 min in the dark. Absorbance was measured at 517 nm against a blank using a spectrophotometer. All experiments were performed in triplicate. The percentage of antioxidant activity was calculated according to the following formula: DPPH scavenging (%) = [(A DPPH − A sample)/A DPPH] × 100.

#### 2.7.2. Thiobarbituric Acid (TBA) Assay

A thiobarbituric acid (TBA) assay was carried out to evaluate the lipid peroxidation inhibition activity of the RV samples. The assay is based on the reactivity of an end product of lipid peroxidation, malondialdehyde (MDA), with TBA to produce a pink adduct (TBA-MDA-TBA). The adduct formed is measured at 535 nm using a spectrophotometer. 

Specifically, the activity of RV towards the oxidation of linoleic acid was determined. For this purpose, the RV samples (RV coarse suspension, RV Phytosome^®^ suspension, RV-NC and LC RV-NC nanosuspensions) were added to a linolenic acid dispersion (1% *w*/*v*) in a 4% *w/v* SDS aqueous solution. Peroxidation was then initiated by the addition of 100 μL of 2 mM ferric chloride and incubation at 37 °C for 30 min. Subsequently, an aliquot of the samples was withdrawn and subjected to a TBA assay. Linoleic acid peroxidation was evaluated in the absence of the RV samples as a control.

For the TBA assay, the sample (0.2 mL) was placed into a glass tube that was closed with a screw cap, and 0.1 mL of water, 0.2 mL of 4% *w/v* SDS, 1.5 mL of 1% *w/v* phosphoric acid and 1.0 mL of 0.6% *w/v* TBA were added. The mixture was stirred and heated in a water bath at 95–100 °C for 45 min to favor the formation of the complex. After cooling in an ice bath, 4 mL of 1-butanol were added to each tube and the TBA–MDA–TBA complex was extracted via stirring and centrifugation. The absorbance of the supernatant that contained the TBA–MDA–TBA complex was read at 535 nm. The lipid peroxidation inhibition (% LPI) was calculated according to the following formula: (% LPI) = [(A control − A sample)/A control] × 100.

### 2.8. In Vivo Pharmacokinetic Study after Oral Administration to Rats

An aqueous nanosuspension of lipid-coated *trans*-resveratrol nanocrystals (LC RV-NC) was prepared for in vivo administration, via the dispersion of the NC powder in water. A suspension of RV was made via grinding the coarse RV powder in a mortar and then suspending it in water that contained hydroxypropyl cellulose (0.5% *w*/*v*), and a commercial RV Phytosome^®^ was used for comparison purposes. Eight-to-ten-week-old male Wistar rats of 250 g were employed. The RV samples were administered directly into the duodenal lumen of fed rats at a concentration of 10 mg RV/mL. Five awake rats with a surgically implanted duodenal cannula were used for each formulation. This administration method was already described and validated [30]. The volume administered was 1 mL of each formulation to achieve a RV dose of 10 mg/rat (40 mg/kg). Blood samples were collected, through another surgically implanted cannula in the jugular vein, into heparinized tubes at designated times up to 24 h after administration. The procedures conformed to the institutional guidelines on animal welfare of the Ethics Committee of the University of Turin (D. Lgs. 26/2014 implementation of directive 2010/63 UE) as well as international guidelines, with all effort being made to minimize the number of animals and their discomfort (3R guidelines). All experiments on animal models were performed according to an experimental protocol that was approved by the University Bioethical Committee and authorized by the Italian Ministry of Health (authorization n. 0165/2015).

### 2.9. Statistical Analysis

All experiments were conducted in triplicate and data are presented as means ± standard deviation (SD). Statistical analysis was performed using GraphPad PRISM version 8.0 software (San Diego, CA, USA). Statistical significance was determined using the non-parametric Student’s *t*-test. 

Statistically significance was reported for *p* value < 0.05. The maximum plasma concentration over the time span (C_max_), the time point of maximum plasma concentration (T_max_) and area under the plasma concentration–time curve (AUC) were calculated using Kinetica^®^ software (Thermo Fisher Scientific Inc., Waltham, MA, USA).

## 3. Results and Discussion

Resveratrol shows relevant therapeutic potential thanks to its antioxidant, cardioprotective, anti-inflammatory and anticancer properties. However, RV’s in vivo efficacy is hampered by its physico-chemical (low solubility and photo-instability) and pharmacokinetic (rapid metabolism and elimination and poor bioavailability) features [31]. This work investigates the feasibility of producing lipid-coated *trans*-resveratrol nanocrystals to overcome the limitations of the molecule and improve its antioxidant activity. The formulation rationale was to merge two technological approaches, nanocrystals and lipid-based systems, that are currently used to increase the oral bioavailability of poorly soluble drugs.

RV nanocrystals were prepared in a purposely tuned milling method. The milling process is a top-down manufacturing technology that is widely used in the industry for particle-size reduction, as found in FDA-approved products [32,33].

Coating with a lipid compound, i.e., phosphatidylcholine, was then performed to favor nanocrystal dispersion in water and the oral absorption of RV after dissolution. Following the dispersion in water of LC RV-NC, a homogeneous colloidal nanosuspension was obtained.

Table 1 reports the physico-chemical characteristics of the two RV nanocrystal formulations, uncoated and coated with phosphatidylcholine, and sizes below 500 nm and a rather homogenous size distribution were observed for both nanocrystal formulations. The presence of phosphatidylcholine during the second milling step produced smaller particle sizes than for RV-NC. Indeed, a size reduction of about 30% was shown. These results were confirmed by SEM analysis. 

**Figure 2:**
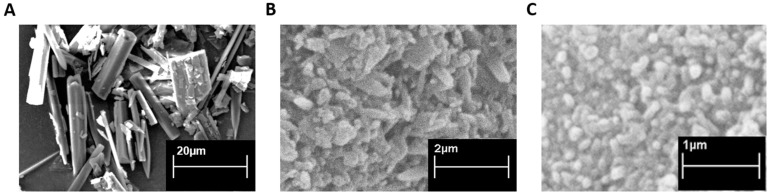
SEM images of raw resveratrol (**A**), *trans*-resveratrol nanocrystals (**B**) and lipid-coated nanocrystals (**C**).

SEM images of raw RV and the lipid-coated and uncoated RV nanocrystals are shown in Figure 2. The raw mortar-ground RV powder displayed needle-shaped morphology, with a mean particle size of about 30 µm and a wide particle size range, whereas the NC displayed smaller sizes and more rounded morphology. Moreover, the lipid-coated NC had a more spherical shape and smoother surfaces than the uncoated analogues. After the milling step, both of the NCs had a narrow size distribution. As shown in Table 1, while RV-NC had a weakly negative surface charge, LC RV-NC showed a Zeta potential value of about −25 mV. The negative Zeta potential of lipid-coated NC can be ascribed to the phosphate groups of phosphatidyl choline on the surface of NC. It is worth noting that the nanoparticle surface charge is a critical factor that affects the physical stability of a dispersed system, since the strong electrostatic repulsion between highly charged nanoparticles prevents them from aggregating [34,35].

**Figure 3:**
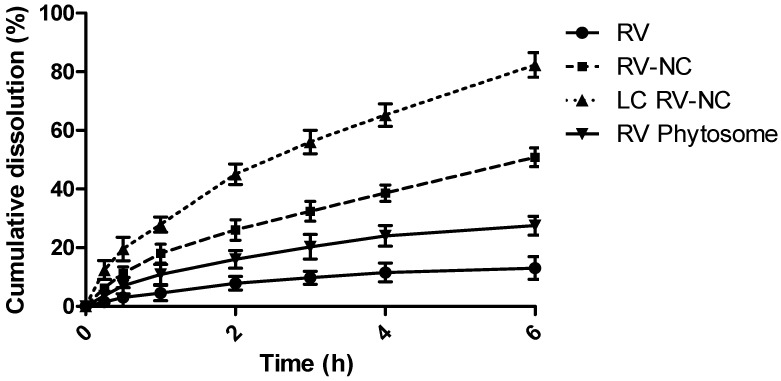
In vitro dissolution profiles of the *trans*-resveratrol NCs (RV-NC) and lipid-coated *trans*-resveratrol NCs (LC RV-NC) in comparison with the coarse resveratrol powder (RV) and RV Phytosome^®^.

The in vitro dissolution profiles of RV-NC and LC RV-NC are reported in Figure 3. For the in vitro dissolution studies, all the RV formulations were suspended in simulated intestinal fluid. The nanosized RV had a faster dissolution rate than the coarse RV powder. In particular, the cumulative dissolution rate % was around four-fold higher for the RV nanocrystals than for RV. An even higher (about 6.3-fold) dissolution rate was observed for the lipid-coated nanocrystals.

The enhanced dissolution rate was caused by the increased surface area, which was, in turn, caused by the marked reduction in particle size, from the micron to nanometer scale. The high surface area and increased saturation solubility are the key parameters for the significant improvement in the dissolution rate of NC [36,37]. In this paper, the highest dissolution was observed for the lipid-coated nanocrystals. Indeed, they were smaller in size than the uncoated ones. In addition, coarse drug particles offer high surface resistance due to their hydrophobic nature, which inhibits them from dissolving into the dissolution medium. On the other hand, adsorbed phosphatidylcholine may reduce drug surface tension in the medium, improving its dissolution [38]. The slower dissolution rate of RV Phytosome^®^ than that those of NC formulations is related to the presence of particles with greater sizes and with lower wettability properties.

**Figure 4:**
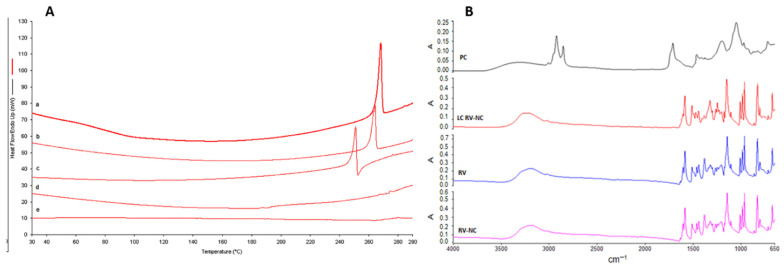
(**A**) Differential scanning calorimetry (DSC) thermograms of (a) *trans*-resveratrol (RV), (b) RV nanocrystals (RV-NC), (c) lipid-coated RV nanocrystals (LC RV-NC), (d) RV Phytosome^®^ and (e) phosphatidylcholine. (**B**) FTIR spectra of the coarse *trans*-resveratrol powder (RV), *trans*-resveratrol nanocrystals (RV-NC), lipid-coated *trans*-resveratrol nanocrystals (LC RV-NC) and phosphatidylcholine (PC).

The characteristics of the RV solid formulations were then studied via thermal analysis and FTIR spectroscopy (Figure 4). The DSC profiles of the coarse RV powder and RV nanocrystals are reported in Figure 4A. The endothermic peak at about 265 °C in the DSC thermogram of the coarse RV powder is related to the fusion of RV (curve a). Following the milling process, a decrease in the melting temperature of RV-NC was observed (T_m_ 262 °C). The presence of a lipid coating on the NC surface is evidenced in the thermogram of the LC RV-NC (curve c) by a further decrease in the melting temperature (T_m_ 248 °C). On the contrary, the DSC thermogram of the RV Phytosome^®^ displayed almost the disappearance of the melting endothermic peak of *trans*-resveratrol at 265 °C, confirming the interaction between RV and phosphatidylcholine in the solid dispersion. Figure 4B shows the infrared spectra of the coarse *trans*-resveratrol powder and those of the uncoated and lipid-coated *trans*-resveratrol nanocrystals for comparison. The FTIR absorption of RV showed the characteristic intense bands between 1400–1300 cm^−1^ and 1700–1600 cm^−1^, which correspond to C–O stretching and C–C aromatic double-bond stretching, respectively. Moreover, an absorption band at 3300 cm^−1^, which corresponds to OH stretching, was observed. The crystalline pattern of RV was not modified by the milling process. However, lipid-coated RV-NC showed shifts and disappearance of some peaks. These changes in their spectral features, compared to RV, indicated that RV–lecithin interactions may occur.

The LC RV-NC formulation was stable overtime for up to 6 months. Indeed, no significant size increase was observed (data not shown). Surface stabilizers hold a crucial role in promoting the physical stabilization of nanocrystals. The agglomeration of the nanocrystals in an aqueous suspension can occur, as can the Ostwald ripening phenomenon [39]. Ionic (i.e., sodium dodecyl sulfate and sodium cholate) and non-ionic surfactants or polymers (i.e., polyvinyl alcohol, polyvinylpyrrolidone, polysorbates, pluronic, poloxamers and cellulose polymers) can be employed to stabilize NCs in aqueous nanosuspensions [40]. Ionic surfactants and charged polymers can prevent aggregation via electrostatic repulsion, while non-ionic surfactants and non-charged polymers can provide a steric barrier around the particles, preventing aggregation [41]. Here, soybean lecithin was selected for NC stabilization as it is a well-known dispersant and surfactant, and it is widely used as a pharmaceutical excipient, being FDA-approved, safe, non-toxic and biocompatible [42]. The lipidic coating favored RV nanosuspension physical stability by means of steric and ionic stabilization. In addition, lecithin had a role in decreasing the Zeta potential to a negative value that was high enough to avoid the NC aggregation phenomena.

**Figure 5:**
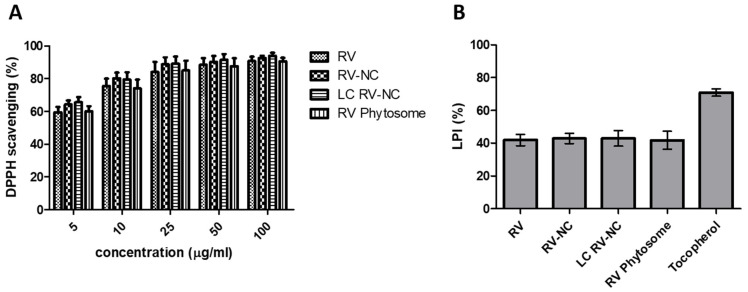
(**A**) In vitro antioxidant activity of *trans*-resveratrol nanocrystals (RV-NC), lipid-coated *trans*-resveratrol nanocrystals (LC RV-NC), RV Phytosome^®^ and the coarse RV suspension (RV) at different concentrations, expressed as DPPH radical scavenging percentage. (**B**) Lipid peroxidation inhibition activity of *trans*-resveratrol nanocrystals (RV-NC), lipid coated *trans*-resveratrol nanocrystals (LC RV-NC), RV Phytosome^®^ and the coarse RV suspension (RV), evaluated in a TBA assay. The experiments were conducted in triplicate and data are presented as means ± standard deviation (SD).

The beneficial health effects of RV are highly associated with its antioxidant activity [43]. The in vitro antioxidant activity of lipid-coated and uncoated RV-NC was evaluated by determining DPPH free radical scavenging activity in comparison with a coarse RV suspension (Figure 5A). After the interaction with a free radical quencher, the strong violet color of the DPPH radical in solution fades to either colorless or pale yellow. The extent of the color decrease, measured as the reduction in absorbance, represents the scavenging efficiency of the samples [44].

RV demonstrated its high antioxidant activity as it displayed increasing DPPH scavenging percentage with increasing RV concentration. The particle size reduction process did not impair RV’s antioxidant properties. In fact, the results show that the scavenging ability of RV-NC was greater than that of the pure resveratrol water suspension. The lower inhibition of the DPPH free radical by the coarse RV may be due to its poor water solubility. These results suggest that the milling step did not induce the *cis*/*trans* isomerization of RV. In addition, the inhibitory activity of RV towards the peroxidation of linoleic acid was evaluated in a TBA assay, and was compared with a common antioxidant, α-tocopherol. Both RV-NC showed a similar capability to inhibit lipid peroxidation. The inhibitory activity of the RV samples was lower than that of tocopherol.

**Figure 6:**
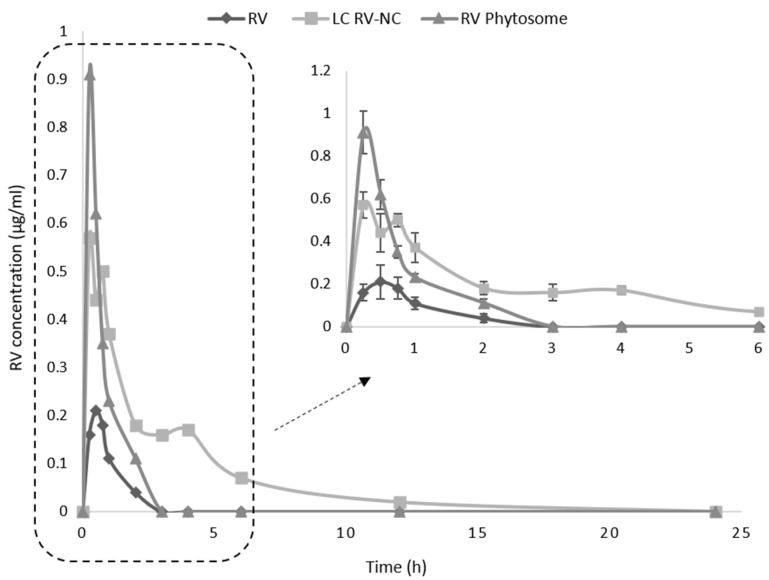
Plasma concentrations of *trans*-resveratrol vs. time after the oral administration of RV coarse powder (RV), lipid-coated RV nanocrystals (LC RV-NC) and RV Phytosome^®^.

The in vivo pharmacokinetics of LC RV-NC was evaluated and compared to the coarse RV suspension after oral administration to rats. Moreover, a RV, Phytosome^®^, which is currently on the market, was evaluated for comparison purposes. The RV dose administered to rats in the in vivo pharmacokinetic study corresponded to 40 mg/kg. It has been reported that RV displays a good safety profile with no toxicity up to the dose of 3 g/kg/day in rats [45]. The results show that LC RV-NC had longer-lasting RV concentrations in the plasma than the other two formulations (Figure 6). 

The pharmacokinetic parameters of the RV formulations after oral administration to rats are reported in Table 2 and roughly six- and three-fold increases in AUC were observed for RV lipid-coated nanocrystals and RV Phytosome^®^, respectively, in comparison to RV. The higher AUC observed can be correlated to the increased in vitro dissolution rate of the NCs, as already shown [46,47]. This behavior is consistent with the BCS classification of RV in class II. Indeed, the increase in apparent drug solubility produces an oral bioavailability improvement as far as class II drugs are concerned [48]. Increased solubility results in a higher concentration gradient at membranes, which leads to improved penetration and permeation through them. Moreover, NC have mucoadhesion capabilities that would favor adhesion to the gastrointestinal mucosa. As a consequence, the prolonged retention of NCs can generate a higher concentration gradient across the gastrointestinal tract, improving drug absorption and hence its bioavailability [49]. Interestingly, the mean residence time (MRT) of LC RV-NC was about three-fold higher than those of the other two RV formulations.

The influence of the different types of formulation on RV oral bioavailability has previously been observed in different animal models [50,51]. For example, Singh et al. have shown that an optimized nanocrystal formulation that was orally administered to rats displayed a significant increase in AUC (3.5-fold) and C_max_ (2.2-fold) in comparison with the coarse RV powder [25].

Interestingly, the lipid coating can also contribute to enhancing RV oral bioavailability. In previous studies, lecithin-based RV nanoemulsions have proven to be the best formulations in permeability assays on Caco-2 cells, being able to deliver RV through the cell monolayer with permeation times that are shorter than the RV metabolization time [52]. This behavior correlates with the C_max_ value of the Phytosome^®^ formulation.

## 4. Conclusions

In the present work, lipid-coated resveratrol nanocrystals were successfully obtained in a top-down manufacturing method using a milling process. The preparation method is easy, cost-effective and scalable and the size reduction process did not affect the resveratrol antioxidant properties. Nanocrystals showed an enhanced in vitro dissolution rate compared to the coarse powder. The increased and prolonged resveratrol in vivo absorption suggested that the formulation could enhance the *trans*-resveratrol antioxidant activity and oral bioavailability.

## Figures and Tables

**Table 1:** Physico-chemical characteristics of RV nanocrystals (RV-NC) and lipid-coated RV nanocrystals (LC RV-NC). Each reported value is the average of 10 independent measurements of 3 different formulations. The data are presented as means ± standard deviation (SD). * *p* < 0.05; ** *p* < 0.01; *** *p* < 0.001 vs. RV-NC.

	RV-NC	LC RV-NC
Average diameter ± SD (nm)	445.8 ± 35.3	306.2 ± 12.5 ***
Polydispersity index (PDI) ± SD	0.25 ± 0.02	0.22 ± 0.02
Zeta potential ± SD (mV)	−2.12 ± 0.54	−26.63 ± 3.17

**Table 2:** Pharmacokinetic parameters of the coarse *trans*-resveratrol suspension (RV), lipid-coated *trans*-resveratrol nanocrystals (LC RV-NC) and RV Phytosome^®^. The results are expressed as the mean ± standard deviation (SD). * *p* < 0.05; ** *p* < 0.01; *** *p* < 0.001 vs. RV.

Parameters	RV	LC RV-NC	RV Phytosome^®^
AUC (h/ng/mL)	250 ± 20	1570 ± 32 ***	780 ± 26 ***
T_max_ (min)	30 ± 1.4	15 ± 0.8	15 ± 0.6
C_max_ (µg/mL)	0.21 ± 0.03	0.62 ± 0.04	0.90 ± 0.05
MRT (h)	1.10 ± 0.02	3.90 ± 0.08	1.10 ± 0.04

## Data Availability

The data presented in this study are available in the article.
